# Physiology

**Published:** 1861-01

**Authors:** S. Weir Mitchell

**Affiliations:** Lecturer on Physiology in the Phila. Med. Association


### PHYSIOLOGY.

BY S. WEIR MITCHELL, M.D.,

LECTURER ON PHYSIOLOGY IN THE PHILA. MED. ASSOCIATION.

### I.—*On the Physiological and Therapeutical Properties of the Peroxide of Hydrogen.* By Dr. Benj. W. Richardson.

This substance, which was discovered by Thénard in 1818, is, in fact, water charged with oxygen in the active state. In his paper, Dr. Richardson took up the following points: The history of the substance; its preparation with special regard to pharmaceutical applications; its physical and chemical properties; its relations to ozone; its physiological properties; its therapeutical value.

It was obvious from the author's description that some obstacles lie in the way of the use of peroxide of hydrogen for medicinal purposes, owing to the difficulty experienced in its manufacture. This difficulty, however, Dr. Richardson greatly simplified, and we should infer that any experienced pharmaceutist could supply the medicine after a short acquaintance with the process of making it, as readily as quinine or other remedial bodies. In preparing it, time and care are the most important requisites. It was shown, indeed, that if perfectly pure peroxide of barium were supplied to the profession, every practitioner in the country could make his own solution of oxygen as he might want it.

The description of the chemical and physical properties of peroxide of hydrogen was unusually interesting. Passing over the facts relating to the influence of inorganic bodies upon it, those bearing on organic matter strike one most forcibly. Thus blood freed from fibrin absorbs the oxygen from the peroxide, and if it is venous blood it becomes arterial, with a rise in the temperature. Washed fibrin and cellular tissue in the fresh state evolve the oxygen; albumen, urea, gelatin, fibrous membrane, and skin produce no change. Grape sugar, and indeed all the sugars brought in contact with it, become decomposed and evolve carbonic acid. Starch undergoes the same modification.

These observations refer to animal substances recently used; but when putrefaction has commenced, then the oxygen of the peroxide seems to act on all alike, and to produce rapid disintegration.

Another curious fact relating to the peroxide was, that its oxydizing power was easily prevented by the presence of certain bodies having a wide extension of names, but analogous characters. Ammonia, in vapor or solution, tobacco, hydrocyanic acid, solution of aconite, and, in short, all the narcotics that are miscible with water, possess this neutralizing property; the permanency of the result being decided by the physical character of the agent employed.

The section of the paper on the relations of the peroxide of hydrogen to ozone was an interesting one, and indicated a careful study of this debated question. It is clear that Dr. Richardson looks upon the two bodies as one and the same. If he has any doubt, it is to the effect that in peroxide of hydrogen there is not any affinity at all between the two elements, hydrogen and oxygen. We pass the matter over to dwell on the physiological actions of the peroxide. These seem to arrange themselves into the following brief propositions. A weak solution oxydizes blood; but this effect can be stopped by the action of the alkaloids and of narcotics. The peroxide supports the life of fishes; but the body of the animal causes rapid evolutions of the gas. The solution injected into the left side of the heart of an animal restores the irritability, but appears to have an opposite effect on the right side. Injected into the arterial system immediately after death, it seems to restore to the muscles the power of contracting on the application of an irritant. It suspends to a considerable extent post-mortem rigidity, and it reduces spasmodic action, excited by such bodies as ammonia and hydrocyanic acid. On the therapeutical value of this powerful agent, Dr. Richardson did not dwell long; but reserved this essential point for another communication. He showed, however, that as an antidote to the alkaloidal poisons, as an external application to decomposing sores, as an internal remedy in fever, where the patient literally dies from deficient oxygen, and in diabetes, the medicine might be used with the best promises of success. In the way of a pleasant acid drink, one could give, said Dr. Richardson, to the typhus-stricken man, one hundred cubic inches of active oxygen per hour. In diabetes, one fact had been made out also by the author, that under the influence of the peroxide, the quantity of sugar at once became less, and the excretion of urine decreased in a relative degree. After illustrating his paper by experiment, Dr. Richardson concluded by stating that on placing it on the annals of that society, he would guard himself once and for all from any exaggerated suggestion as to the value of this new remedial agent. The subject, indeed, was so novel, that after twelve months study of it, he had feared to use a sentence that had been considered over and over again. He did not pretend to know all the properties of the peroxide. He did not bind himself invariably to any opinion offered on this present occasion; nay, experience might show that the substance discussed, in a medicinal point of view, took new and even different directions from those with which he had opened the argument. His own intentions and objects would be served if he did but call forth investigation and fact, let the course of events bend in whatever way they might.

Dr. Thudichum expressed an opinion that the peroxide of hydrogen, as made by Thénard's process, could not be altogether freed from chlorine, and suggested that the substance should be made by the simple sulphuric acid process. He also dwelt on the fermentation theory in relation to certain of the effects of the peroxide. He expressed a disbelief as to the existence of "ozone."

Dr. Lankester asked Dr. Richardson whether oxygen, as it was liberated from the solution, was in the active state, and Dr. Richardson having answered in the negative, Dr. Lankester proceeded to defend the ozone theory. He believed with the author of the proposition, to use the peroxide of hydrogen as a medicine, that great therapeutical effects would come from it. He suggested experiments to determine whether the amount of excreted matters—such as urea and carbonic acid—would be influenced by the administration. It was a remarkable fact, that so powerful a substance should so long have remained uninvestigated as a medicinal agent.

Dr. Garrod compared the chemical effects of the peroxide of hydrogen with the effects of the permanganates. He had tried the permanganates in diabetes, but he thought only with the effect of increasing the amount of sugar. He would wish to hear whether the physiological effects of the peroxide and of a permanganate were the same. It might be that the saline character of the latter produced a difference.

Dr. Richardson, in reply, said that in regard to the presence of a trace of chlorine in the solution, it made no difference; for the effects of chlorine-water were very analogous, and it might turn out ultimately that a direct relationship could be traced between chlorine and oxygen. Thus water, boiled free of oxygen, and charged very feebly with chlorine, would, as he should show on another occasion, support the life of a fish longer than the water altogether destitute of oxygen. In respect to ozone, he had no doubt that such a substance existed, and he was inclined to think that it was a modification of oxygen. He thought that in testing the effects of the peroxide of hydrogen, it were best to try it in disease without any further intermediation, selecting for its trial extreme diseases, such as were not at this time amenable to treatment. The actions, *physiologically*, of the peroxide of hydrogen and of the permanganates were entirely different; the latter, injected into the veins of a horse, produced permanent fluidity of the blood, and transformed the blood on the arterial side to the venous color. The difference depended on the fact that in the permanganate the oxygen was combined, while in the peroxide it might be considered as virtually free.

Dr. Garrod, Dr. Radcliffe, and several other Fellows observed that they should be anxious to give the peroxide a fair trial as to its therapeutical value; and inquiry was made as to the means of obtaining the solution.—(*Lancet*, October 20, 1860.)

### II.—*On the Functions of the Cœliac and the Mesenteric Plexus.* By PROFESSOR BUDGE.

Numerous experiments of extirpation of the cœliac ganglia and the mesenteric ganglion were performed on rabbits. The animals generally died within twenty-four hours after the operation; in a few cases death did not occur till the third day. The effects of the operation, briefly reviewed, are: 1. Softening of the fæces, so that frequently no consistent mass is found in the rectum, but only a completely soft mash. 2. Increased motion of the bowel. 3. Elimination of tough, glairy mucus and blood. 4. Enlargement of the liver. 5. Manifestation of pain and suffering, unless the splanchnic nerve had been previously cut.—(*Nova Acta Acad, caes. Leop.—Car. Nat. Cur.*, xix. pp. 257–284, 1860; *American Medical Monthly*, October, 1860.)

### III.—*A Contribution to the Chemistry of the Supra-renal Capsules.* By DR. SELIGSOHN.

The following experiments were undertaken in the Laboratory of the Pathological Institute, at Berlin, with a view to establish the discovery by Vulpian and Cloëz (Comptes Rendus, September, 1857, p. 340,) of taurocholic and hippuric acids in the supra-renal capsules. The method pointed out by these observers was adopted in my investigations.

A pound of the supra-renal capsules of the ox having been carefully cleaned and comminuted, was macerated for several days with diluted alcohol, after which the alcoholic solution was filtered and evaporated. The addition of water to the completely evaporated mass caused the separation of fatty matters. The watery solution was evaporated to the consistence of a syrup, and absolute alcohol was added. The residuum thus obtained was dissolved in water to be tested for leucin; the precipitate produced in the red-colored solution, by the addition of basic acetate of lead, was separated by filtration, and the excess of lead in the filtrate was removed by sulphuretted hydrogen. In the fluid freed by filtration from sulphuret of lead, and evaporated to the consistence of a syrup, only cubic crystals (NaC) were observed on miscroscopic examination, but no leucin was discovered. The alcoholic solution filtered from the supposed residuum of leucin was evaporated to the consistence of a thick, brown syrup, which, when dissolved in water, manifested an acid reaction. This solution, mixed with an excess of hydrated oxide of lead, (to the removal of the acid reaction,) was anew evaporated to dryness; the dry mass was then boiled with 65 per cent. of alcohol. From the warm filtered alcoholic solution, which was of a dark-brown color, the excess of lead was removed by means of sulphuretted hydrogen; from the yellow fluid separated by filtration from the sulphuret of lead, crystals were deposited after long repose in the cold, which exhibited the following behavior toward reagents. They dissolved readily in ether, and, on evaporation of the menstruum, separated partly in the form of fine needles, partly as scales. They dissolved with difficulty in cold water, which, however, exhibited a distinctly acid reaction. Under the microscope the crystals presented the form of fissured rhomboidal tables; some of them having been warmed in a test-glass with concentrated hydrochloric acid, the crystalline coating which formed on the sides of the glass exhibited the same form as the original crystals.

Their microscopical and chemical behavior, as well as the formation of the sublimate, put it beyond a doubt that crystals of benzoic acid had in this case separated, while the properties of the crystals obtained by Vulpian and Cloëz, by a similar mode of proceeding, agree essentially with those of hippuric acid.

To the fluid separated from the crystals, an excess of powdered chalk was added, to prevent the possibility of decomposition, (on evaporation in the water bath before the addition of carbonate of lime, the fluid became turbid, while fumes of sulphuretted hydrogen at the same time escaped,) and the former was thereupon evaporated to dryness in the water bath. The dry residuum was strongly warmed with diluted alcohol, the alcoholic filtrate again evaporated, and then heated with concentrated hydrochloric acid, in order to resolve any taurochloric acid, which might have been present, into tauric and choloidinic acid—the crystalline mass thus obtained dissolved with the formation of a residuum apparently agreeing with choloidinic acid; which, however, on the application of Pettenjoker's test, did not exhibit the characteristic coloration. But in the solution the presence of sulphur was clearly demonstrated; moreover, the characteristic crystals of taurin separated on evaporating the solution in the air.

The incinerated constituents of the residuum of the supra-renal capsules obtained after maceration in alcohol, in which no uric acid was discovered, were:—

1. Constituents soluble in water:—

Phosphate of potash.
" " soda.
" " lime.

2. Constituents soluble in hydrochloric acid:—

Phosphate of iron.
" " lime.
" " magnesia.

With reference to the color reaction of the supra-renal capsules, I would, in conclusion, observe that after the application of very dilute hydrochloric acid to their substance, the filtered clear solution assumes a beautiful red color on the addition of ammonia in excess; after the evaporation of the excess of ammonia the solution again becomes colorless.—(*Virchow's Archiv*, Band xviii., 3 and 4 Heft, p. 355, and *Am. Med. Monthly*, October, 1860.)

IV.—*Presence of Sugar in the Liver.* By DR. HARLEY.

Dr. Harley has re-examined the subject of the presence of sugar in the blood and the liver. He disputes entirely Dr Pavy's conclusions, and, with slight exception, agrees throughout with M. Bernard. It will be remembered that Dr. Pavy attempted to show that the existence of sugar in the liver was due to post-mortem changes. To prove this he froze parts of the livers of animals instantly after death had been caused by pithing, intending thus to suspend the action of the causes which he supposed to be active after death and to give rise to the production of sugar. The parts which remained unfrozen were found to contain sugar, those which had been promptly frozen contained none.

Dr. Harley gives in detail the experiments which he made when following throughout Dr. Pavy's plan. He arrived at quite opposite conclusions, however, and ascertained most positively that the production of sugar is not a post-mortem result. Dr. Thudichum had already contradicted another portion of Dr. Pavy's results, and had shown one of the fallacies which had led that chemist astray. We quote in full the latter part of Dr. Harley's paper which contains his general conclusions and his objection to one of the opinions of M. Bernard.

"The only point now remaining was to determine quantitatively the increase in the amount of sugar in the liver after its removal from the body, and for that purpose we preferred operating on an animal fed on a mixed diet.

"Experiment 7.—A small dog, which had been previously fed on an animal diet, received a full meal of bread and milk. Five hours after, the animal was killed, and a portion of the liver rapidly sliced off, and immersed in a freezing mixture. A ligature was placed on the portal vein, and its blood collected before the circulation had ceased. On examination, this blood was found to contain a small quantity of sugar, derived no doubt from the food. Bernard, I believe, has erred in supposing that all the saccharine matter found in the animal organism is formed out of the glucogen produced in the liver. This no doubt is the case in the carnivora, where the diet is restricted to food inconvertible into sugar in the alimentary canal, but cannot be regarded as the natural state of things either in the omnivora or herbivora; for the food of the latter not only contains sugar, but its amylaceous elements may be converted into that substance in the process of digestion. The sugar found in the bodies of animals fed on a mixed diet ought therefore to be regarded partly as the direct product of the food and partly as derived from the glucogen formed in the liver.

"Bernard's chief argument against this view is founded on the fact that the livers of dogs fed on a mixed diet contain no more sugar than those fed on purely animal food. In my opinion, however, this fact is not sufficient to decide the question; for as the liver does not store up sugar, the quantity it at any time contains is no criterion of the amount produced in it. Moreover, the sugar derived from the food need not be expected to be found in the liver. Had Bernard gauged the sugar present in the blood, instead of that in the liver, after each kind of diet, the result obtained would, I believe, have led him to a different conclusion. This being a point of great practical importance in the treatment of diabetes, I may be here permitted to mention that I have occasionally found nearly twice as much sugar in the blood of an animal using a mixed diet as in that of one feeding on flesh alone.

"To return to the last experiment: about two hours after the death of the animal, portions of the frozen part of the liver, and of that which had been kept warm in the body of the animal, were carefully weighed, and the proportions of sugar they respectively contained estimated by volumetric analysis.

"The portion of frozen liver was found to contain 0·333 per cent., and that of the other 1·55 per cent. of saccharine matter. It is thus seen that in two hours the sugar in the liver had augmented nearly fivefold. As Bernard has shown, the simple washing out of the liver, by passing a stream of water through its vessels, would remove all the sugar anteriorly formed. On placing it again aside for a short time, a fresh portion of sugar would form in it at the expense of the glucogen: 0·333 per cent. of sugar seems a small quantity; but if we suppose a liver weighing, as in man, not less than fifty ounces, to contain 0·333 per cent., above seventy grains of sugar would be present in it at the moment of death—no very insignificant quantity, when it is recollected that sugar is removed from the liver at every pulsation of the heart, to be partly consumed, and that it is as continually supplied by the organ.

"The results of the experiments now related do not therefore in any way countenance the notion that sugar is not produced in the healthy animal body. On the contrary, such conclusions as they afford are altogether in favor of the generally received views upon the subject.

"From the preceding experiments the following conclusions may be drawn:—

"First. Sugar is a normal constituent of the blood of the general circulation.

"Secondly. Portal blood of an animal on mixed diet contains sugar.

"Thirdly. Portal blood of a fasting animal, as well as of an animal fed solely on flesh, is devoid of sugar.

"Fourthly. The livers of dogs contain sugar, whether the diet is animal or vegetable.

"Fifthly. Under favorable circumstances saccharine matter may be found in the liver of an animal after three entire days of rigid fasting.

"Sixthly. The sugar found in the bodies of animals fed on mixed food is partly derived directly from the food, partly formed in the liver.

"Seventhly. The livers of animals restricted to flesh diet possess the power of forming glucogen, which glucogen is at least in part transformed into sugar in the liver—an inference which does not exclude the probability of glucogen (like starch in the vegetable organism) being transformed into other materials besides sugar.

"Eighthly. As sugar is found in the liver at the moment of death its presence cannot properly be ascribed to a post-mortem change, but is to be regarded as the result of a natural condition."—(*Lancet*, September, 1860.)

### V.—*On Muscular Poisons.* By M. CLAUDE BERNARD.

There exist other poisons which equally destroy the vital properties of the locomotive apparatus, although a totally different histological element becomes the seat of disorganization. We allude to the toxic agents which abolish contractility in the muscular tissue; you will find them infinitely more numerous than those which confine their action to the nerves. Among this class of bodies, digitalis and upas antiar* have already been mentioned; and the attention of American physiologists has lately been drawn by Drs. Hammond and Mitchell, of Philadelphia, to the properties of two other substances which are known by the Indian names of Corroval and Vao. Their action is similar to that of upas, although of greater intensity. The active principle of the veratrum album or veratrine, a substance now frequently employed in practice, also exerts its influence upon the muscular fibre, to the exclusion of all other tissues; and a large number of poisons, with the chemical composition of which we are imperfectly acquainted, evidently belong to the same class.

The small arrows now placed before you were forwarded to us by M. Boussingault. These weapons are extensively used in South America. The nature of the substance with which they are impregnated is still a mystery to us; they are supposed to have been charged with woorara, but their action is of a totally different kind; the muscular, not the nervous, fibres are affected by the wounds which they inflict. Although unable to state in a positive manner what this poison really is, I am tempted to believe that the venom of toads is the substance employed. The Indians of New Grenada are known to make use of a small variety of this animal, which is found in great abundance in that country, for the purpose of poisoning their weapons. After collecting a large number of toads, they impale them upon wooden spits and roast them alive. The heat causes the animal to eject its venom, in which the arrows are dipped. This latter substance is known in fact to exert a most powerful action on the muscular tissue. But the unknown principle with which these arrows are impregnated differs so completely in its properties from those usually attributed to animal poisons, that we shall seize the present opportunity to enter into some considerations on the subject of venoms in general, and that of the toad in particular, our conclusions being the result of the researches we have lately undertaken on this interesting and hitherto obscure part of toxicology.

---

* There exists another kind of upas, which is merely a preparation of strychnia.

The effects produced on animals by these poisoned arrows would appear at first sight to be strictly similar to those produced in our own climate by the venom of toads; but when we inquire into the chemical composition of this new substance, we find it to be a perfectly stable compound, the properties of which are not destroyed by plunging it into boiling water or dissolving it in alcohol. The arrows which have been allowed to rest for a certain space of time in this latter solvent, are found to have lost their destructive power, although no change has taken place in their dark-brown color; and the liquid in which they have been kept being gradually evaporated, the solid residue left behind appears to contain the active principle which imbibes them, for on redissolving it in water, a poison is obtained which produces on animals the same effects as the arrows themselves.

But the opinions usually entertained with respect to venoms are entirely opposed to such a result. Animal poisons are in general considered as resembling ferments in their chemical properties, being easily destroyed by the action of heat, and modified by dissolution in alcohol and other powerful solvents. We ought, therefore, in accordance with generally received opinions, to have surrendered our former hypothesis; but however satisfactory the inductions drawn from previously ascertained facts may appear, an experimental verification is always indispensable in sound physiology, even in those cases in which it seems useful and almost absurd to require experimental proofs. Acting in conformity with this rule, which has always directed us up to the present time in our labors, we have examined in its turn the venom of toads for the purpose of ascertaining whether its characteristics were really such as had been supposed, and, contrary to our expectations, we have found it to be soluble in alcohol, and in all other respects as stable a compound as the active principle of these American arrows, for the action of boiling water destroys none of its properties.

It had been supposed, however, that no venom was fatal to the animal itself which secreted it; and as the poisonous arrows produce marked effects upon the toad, we found it necessary to try upon this animal the results produced by the inoculation of its own venom, in order to ascertain whether the opinion generally entertained on this point was scientifically true. The experiments undertaken for this purpose do not allow the slightest doubt to remain on this point. The toad is killed, like other animals, by the inoculation of its own venom; it is, however, true that the action of this poison is infinitely less intense in toads than in frogs, of all other animals those which resemble them most in organization; and it might hence be inferred that a certain degree of truth exists in the above-mentioned opinions. Let us bear in mind, however, that the resistance of the toad to all kinds of poison is infinitely superior to that of the frog; that strychnia, upas, and various other substances act in much smaller doses upon the latter than upon the former animal—a fact which agrees with the lower excitability of the nervous system in toads.

All these phenomena may therefore be reduced to a general principle, which we have strenuously endeavored to inculcate in the course of the present lectures, namely, that no essential differences exist between the homologous tissues of different animals; that properties observed in one case are found to exist throughout the entire scale of being, although considerable variations in their intensity are met with even in the case of neighboring species. You also perceive that our knowledge of venoms is less advanced than the other branches of toxicology, and that it is urgent to repeat a new series of experiments on each of those substances in particular; for we are not prepared, up to the present moment, to attribute the properties enjoyed by the venom of toads to the whole series of animal poisons; further researches are indispensable to settle the question.

Let us now, gentlemen, after this digression, resume the explanation of the effects produced by muscular poisons. The principal result of their action is sudden arrest of the heart's motion; and in this respect they might also be divided into two classes. Some of them act upon the heart before affecting the voluntary muscles; such is the case with digitalis and upas antiar. Corroval and vao enjoy this power in a still greater degree. The reverse is the case with other poisons; they act upon the voluntary muscles at first, and do not paralyze the heart till a later period of the destructive process; the American arrows belong to this latter class. It is therefore easy to conceive how wide is the difference between the intensity with which these poisons act in animals differently organized. A very small dose of corroval produces instant death in birds; a mammal survives a few minutes; while frogs resist the action of this substance for a considerable space of time, these animals, as you are aware, being able to survive a few hours after the total ablation of the heart.

We shall now perform a few experiments on various animals with these different poisons, and you will find that far from resembling woorara and strychnia, which destroy life without leaving the slightest vestige of their action, these muscular poisons actually produce deep physical changes in the tissue which loses its physiological properties under their influence.

(At this stage of the lecture a couple of pigeons were brought forward; an incision was made into the breast of both, laying bare the muscular fibres, and on applying test-paper, a decided alkaline reaction was met with in both cases. One of the pigeons was then killed with a poisoned arrow, the other with a solution of woorara injected into the cellular tissue. A frog was at the same time poisoned by introducing a small dose of corroval under the skin; it was then replaced in the jar from which it had been taken.)

You perceive, gentlemen, that the muscular tissue offers a decided alkaline reaction in the healthy state. But in animals poisoned with any of those substances which act upon the contractile elements, the reaction of this tissue becomes acid, and the *rigor mortis* occurs immediately after death; two changes which spontaneously take place in dead animals, but only after a space of twenty-four hours has elapsed. The electrical properties of this tissue equally undergo a singular alteration, for, in the ordinary state of things, the external surface of a muscle is positively, and its internal or cut surface negatively electrified; the reverse is the case in animals poisoned with these toxic agents. And, lastly, on opening the bodies immediately after death, the heart is found contracted, motionless, rigid, and totally empty, so that its transparent walls in the frog have lost the ruddy color imparted by the presence of blood within the cardiac cavities, and appear perfectly white and colorless.

In all such cases the muscular element alone has been acted upon; for if on poisoning an animal with one of these agents, you were to apply a ligature round one of the limbs, and thus prevent the poison from reaching it, you would find that while the other muscles of the body remained insensible to the action of galvanism, those of the limb thus preserved, would readily obey its influence, when excited through the corresponding nerves; a proof that the muscular fibres alone have in this case been interfered with, the nerves retaining as before all their vital properties.

(The bodies of the poisoned animals were now opened; in the pigeon killed with an arrow, the heart was found rigid and motionless, the muscles stiff, and incapable of responding to the galvanic current. The same phenomena were observed in the frog, the heart of which was particularly rigid and perfectly colorless. On testing the muscles, their reaction was found decidedly acid. In the pigeon killed with woorara, the heart was found still in motion; and when at length its pulsations had ceased, galvanism was applied to it, and caused the parietes to contract; the muscles of the limbs responded readily and powerfully to the same agent and their reaction was alkaline.)—(*Medical Times and Gazette*, September 29, 1860.)

VI.—*On Muscular Contractions from Mechanical Irritation in the living Man.* By DR. L. AUERBACH.

Dr. Auerbach demonstrated to the Medical Section of the Schlesiche Vaterländische Gesellschaft, at its session of 1860, three series of phenomena observed by him on striking muscles with a percussion hammer or the finger. The first embraces the instantaneous contraction of the whole muscle when its whole breadth is touched, or of the particular bundle or bundles of fibres struck, when the blow is longitudinally limited. The blow need be but slight, and its effect is apparent to the careful observer, both by sight and on the touch. This Dr. Auerbach regards, not as the result of reflex action, nor of immediate muscular irritability, but as *neuro-muscular* contraction. The second series is one of *idio-muscular* contraction. It can be distinctly seen and felt as a limited hardness or tumor arising from the contraction of the fibres touched, and generally having the form of the instrument used, viz.: while the blunt edge of a knife causes a straight-lined rising or swelling, the touch with a ring causes a ringform hardness, the square base of a hammer, a similarly-formed swelling, etc. The third result, observed from a blow, is a kind of *peristaltic* motion of the obliquely striped muscular fibres, giving the appearance of a wave rolling along the fibres of the muscle, from one end to the other and back again. Dr. Auerbach demonstrated these interesting facts before the section, especially on the biceps muscle of a blacksmith, who had a very muscular arm, and being affected with tuberculosis, was greatly reduced in fat. (Among the practical applications made of these observations, we only refer to the explanation of the benefits resulting from systematic blows on muscles as a means of inciting them to action, as employed in some species of medical gymnastics.)—(*Foriep's Notizen*, III., No. 8, 1860; *American Medical Monthly*, October, 1860.)

Dr. Auerbach has been anticipated in the larger part of what he describes, as new—first by Dr. Stokes, of Dublin, who many years ago recorded, in his work on the chest, the fact that a blow on a muscle causes the whole length of a bar of its fibres to contract; and, secondly, by Dr. S. W. Mitchell, Proceedings Biological Department of Academy of Natural Sciences, Philadelphia, March, 1859. In this latter paper is described, at length, both of the varieties of contractions alluded to by Dr. Auerbach, and, as will be seen from the following extracts, a somewhat different explanation of them is there given.

"Dr. Stokes, of Dublin, long ago observed that when he percussed the skin over the pectoralis muscle, its fibres contracted responsive to the stimulus of the blow. While percussing certain consumptive patients, Dr. Mitchell noticed that as the bar of muscle ceased to contract, a second contraction took place nearly at right angles to the first one. By it the skin was raised into a prominence, some lines in breadth and rather longer than the space covered by the percussing finger end. This second contraction so slowly disappeared that it seemed to be due rather to the action of organic non-striated muscle, than to the striated variety of which voluntary muscles are composed, and which is habitually rapid in its mode of contraction and of relaxation. Further observation, however, showed that a large part of the voluntary muscles, which are neither deeply placed or firmly bound down by fascia, are able to exhibit both of the forms of contraction here alluded to. Thus the extensor muscles of the leg and arm are not very susceptible to this form of direct stimulus, while the flexors and most of the muscles of the trunk, both before and behind, can be made to exhibit both forms of contraction by tapping them smartly and quickly with the finger point or a percussion hammer. The primary contraction, or that which involves the whole length of a fasciculus of muscle, is best seen when we strike upon the region of the pectoralis major or that of the gluteus maximus. The secondary and local contraction is best developed by percussing the pectoral region, and the skin which covers the infra spinatus scapular muscle. Illustrations of the phenomena in question are so frequently within reach of the members that Dr. Mitchell did not consider it necessary to describe them more fully.

"Several circumstances had already convinced Dr. Mitchell that the secondary contraction, described by him, was not due to the action of the non-striated muscle of the skin. A very obvious and simple experimental test at once referred the phenomenon in question to its proper source—the voluntary muscles beneath the cuticle.

"A small rabbit was rendered insensible by the aid of chloroform, and the skin was removed from the chest so as to expose the surface of the pect. major muscle. Upon striking the muscle with a scalpel handle or any blunt body, two distinct reactions ensued: 1. The fasciculus of muscle which was *stretched* by the blow, instantly and rapidly contracted and relaxed. As the relaxation took place, a local contraction occurred at the point struck, so that a small portion of the muscle could be seen to gather itself into a little mound, which again disappeared within from twenty seconds to half a minute. Both phenomena, then, are due to the contractibility of voluntary muscular fibre. Dr. Hammond, who had witnessed the experiment, and who had also seen the phenomenon in question, agreed with the explanation given by Dr. Mitchell."

